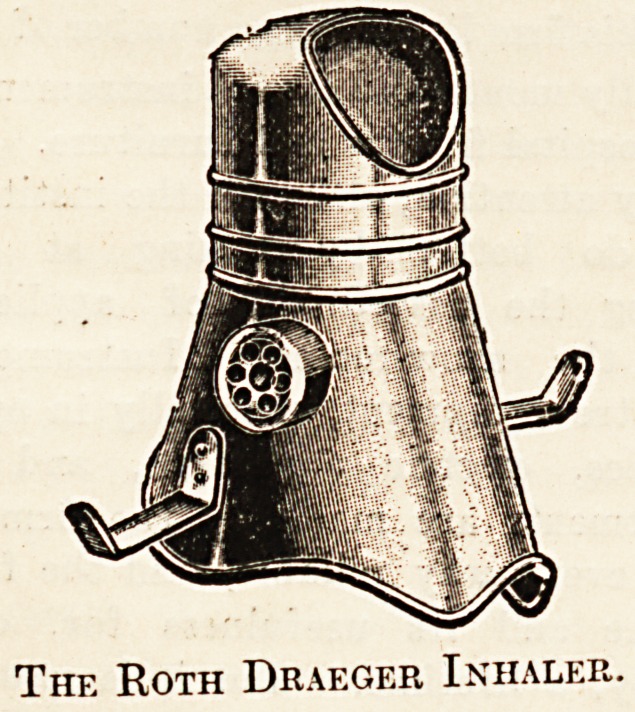# Surgical and InstitutionalAppliances

**Published:** 1911-03-25

**Authors:** 


### SPECIAL SUPPLEMENT.

### SURGICAL AND INSTITUTIONAL APPLIANCES.

### HOSPITAL EQUIPMENT AND INSTRUMENTS FOR THE GENERAL PRACTITIONER.

In the present issue we devote several pages to advertisements by leading firms of hospital furniture and apparatus. It is important that hospital authorities should be in a position to compare and contrast different makes and quotations by various firms; in order to be able to do so it is necessary to consider the different catalogues carefully, and to make due allowance for the advances in construction, alteration in material, and modifications in design, which together mean improvement all round. As it is obviously impossible for us to deal at length with the various firms within the short space at our disposal, we are obliged merely to draw attention to one or two salient details enumerated in their lists in the hope that such cursory indication will prompt hospital workers to give fuller consideration to the extensive catalogues issued by each firm.

A considerable quantity of apparatus of a cheap and inferior quality has for some time past, been placed on the market, and as though price were the paramount feature, this class of article appears to have been readily taken up by hospitals, nursing homes, and institutions generally, where it usually proves a source of trouble and annoyance, owing to its liability to get out of order after a comparatively short time in use.

Institutional workers as well as general practitioners will find it advisable, from time to time, to cast their eyes over instrument makers' catalogues. Progressive development has been enormous during the last few years in the instrument-making world, and it is well that practitioners and hospital authorities should interest themselves in the nature and extent of these improvements, in the alteration of prices, and in the better quality of articles and materials now supplied. In the present issue we give some particulars regarding the firms whose catalogues are specially worth attention, and in the following brief résumé of the facts taken from these price lists are set forth the main items that, owing to their novelty or their excellence, specially call for the practitioners' or the hospital workers' consideration.

#### Some Firms and their Specialties.

Messrs. **ALLEN AND HANBURY'S** manufactures are well known. They are of sterling merit and in every way worthy of the attention of hospital secretaries. We would draw attention to the many improvements listed in their new catalogue. The

The Stack Steriliser.

instrument tables, operating tables, and ward furniture, are all very attractive in appearance and strong in make. One of the novelties listed by the firm is the Stack Steriliser. This a very simple, moderately-priced instrument which should be a part of the outfit of every practitioner who does much surgical work, since it ensures thorough sterilisation of dressings and bandages. The firm also manufactures the instruments required for the performance of Mr. Arbuthnot Lane's special technique in wiring fractures—a description of which appeared in our columns some weeks ago—and Mr. Ryall's Universal Electric Illuminator—an ingenious and adaptable instrument which is very serviceable for endoscopic work. The firm issues special pamphlets regarding Mr. Lane's instruments, in which photographs of cases treated are given.

Messrs. **ROBINSON AND SONS, Ltd.**, of Wheat Bridge Spinning Mills, Chesterfield (London Office, 55, Fann Street, E.C.) are the manufacturers of the well-known "Stag" brand of surgical dressings. Their preparaparations are used in many hospitals, and have been found to give thorough satisfaction. Absorbent cotton, wood wool, wadding and tissue, gamgee tissue, cellulose wadding, towelling, gauze, pads and sponges, and, indeed, all the outfit required for dressing in theatre and ward, are prepared by the firm and listed under the stag mark, which is a certificate of excellence that may be relied upon.

**MABBOTT AND CO., Ltd**, are kitchen engineering specialists, who manufacture apparatus for the economic departments of institutions. They list carving tables and kitchen furniture, potato-slicing and peeling machinery, hot plates, and steam jacketed pans, and a large variety of ovens and ranges. Some of the modifications in the machinery are ingenious, and enable the powerful apparatus to be worked by hand—a matter of considerable importance to small institutions which do not possess the requisite electric or steam power. Every attention has been paid to solidity and strength in the construction of their apparatus, which have won a deserved reputation in those institutions where they have been tried.

**THE NORWICH CRAPE COMPANY, Ltd.**, of St. Augustine's Factory, Norwich, manufactures a very reliable and durable quality of crêpe bandage. All the goods of this firm are made from British-spun yarn, and the company is acknowledged to be the pioneer in this kind of bandage work. The samples of the Norwich crêpe bandage supplied to us fulfil all the requirements of a good-class bandage of the crêpe variety. It is highly elastic, without containing any rubber, and is therefore easily sterilisable by washing. This class of bandage is a necessity in every hospital, particularly in the out-patient department, and the cheapness of the crêpe variety is a great point in its favour.

**THE HOSPITAL AND GENERAL CONTRACTS CO., LTD.**, of Mortimer Street, has on show a very varied assortment of hospital furniture which is well worth inspection. The prices charged by the firm are low in comparison with those of many other houses, but the purchaser may be assured that he is getting the best material at the lowest cost. The specially enamelled ware manufactured by the firm is very durable and easily cleaned and for these reasons particularly suitable for hospital and infirmary use. A large number of modifications of hospital furniture is shown in the firm's lists: some of the models are illustrated in our advertisement columns. The variety of instruments catalogued is equally great, and we would specially refer here to the new sterilisable mouth instruments which the firm has placed on the market. The Hospitals and General Contracts Co., Ltd., is an almost catholic firm so far as hospitals and the practitioner are concerned. It will obtain anything to order; if nothing can be obtained to answer to the purchaser's requirements, it will make the article. Hospital authorities who do not wish the worry and trouble of undertaking ward and theatre equipment themselves can rely upon the firm to do it for them according to the requirements of their institutions.

**Messrs. T. HAWKSLEY AND SON,** of 357 Oxford Street, W., are specialists in clinical investigation apparatus, and many of their specialities are so well known that no detailed description of them is necessary. Gowers' modification of Haymen's Haemocytometer, Haldane's Hoemoglobinometer, Halls' Rotary Haemoglobinometer, varieties of the Riva Rocci apparatus, Bells apparatus for the estimation of calcium salts, and all the other types of apparatus needed for blood examinations are supplied by this firm. The instruments are strongly made, and are at

The Hawksley Viscosimeter—Showing Use.

the same time exquisitely finished, so that their calibration can be confidently relied upon. When furnishing and equipping the institutional laboratory, Hawksley's catalogue should be consulted with regard to apparatus.

A very simple and ingenious form of steriliser is supplied by the **MEDICAL SUPPLY ASSOCIATION,** of 228 Grays Inn Road, W.C. This is MacDonald's steam steriliser for use over fire or gas burner; it needs a very small amount of steam, and all that is necessary when using the steriliser is to pour in a small quantity of water, place in the dressings, adjust the lid, and set the steriliser on whatever heating apparatus is available. Its simplicity and reliability render this one of the best forms of the smaller sterilisers we have seen, and we particularly recommend it to the attention of the cottage hospital secretary. This firm also specialises in hospital fittings and equipment, and list a wide range of new modifications and specialties. The Ross Aseptic Respirator which is made by this firm, is a simple but highly-efficient inhaler for use in respiratory affections.

**Messrs. JOHN WEISS AND SON,** of 287 Oxford Street, W., are well known as makers of first-class surgical instruments and appliances, and their house is historic. They make a specialty of eye instruments, and list the Harman twin scissors for Herbert's operation, and Bradburne's combined tarsal cyst knife and curette. This latter little instrument is a particularly interesting combination which should appeal to the general practitioner on account of its simplicity, and the fact that its use considerably simplifies the small operation for the removal of a tarsal cyst. A large stock of ward furniture of various models is on view in the Oxford Street premises.

**Messrs. WHITE AND WRIGHT,** of Liverpool, are actual manufacturers of hospital furniture, and have recently produced a high-pressure steriliser; it is strongly made, and easy of management. We would also draw special attention to an operation-table, made by this firm, of a heavy pattern, essentially for the hospital theatre. This production is one of the latest in the market, and it is a typical specimen of high-class workmanship. Important features of this table are its perfect drainage and its beautifully free movements. While it rests on very strong

White and Wright's New Model Theatre Operating Table.

brass castors, with rubber tyred wheels, from off which its weight can be instantaneously taken by means of a lever, it is thus rendered "absolutely rigid." It is provided with hot-water tanks, and admits of the most important surgical positions being obtained, including the raising and lowering of the head. This firm also makes a portable operation-table which, from its extreme lightness, rigidity, and excellent finish, is probably second to none. Having workshops on the premises, Messrs. White and Wright are enabled to give personal supervision to all orders entrusted to them. Their manufacture is of a quality calculated to stand the hard wear and tear of the hospital ward. They can be made or modified to any design or from drawings. The firm has usually a large and varied assortment of hospital requisities in stock, at 93 Renshaw Street, Liverpool.

**W. R. GROSSMITH,** of 110 Strand, W.C., forwards a very interesting illustrated catalogue of artificial legs, hands, arms and eyes. This house has paid a great deal of attention to the fitting of artificial limbs, and its appliances may be relied upon to secure the essentials which are so often overlooked by many instrument makers—strength combined with lightness, durability, a wide range of movement with the necessary firmness, and perfectly accurate fitting. The catalogue gives specifications of all the appliances supplied, and a detailed scheme for the measuring of patients requiring artificial limbs. The firm does contract work, and supplies special quotations for special work, deformities and unusual amputations. A practitioner who is unwilling to send a patient to an instrument maker, who so often takes entire charge of the patient to the exclusion of the practitioner, cannot do better than consult Mr. Grossmith's catalogue and deal with the case himself after a study of the essentials as laid down in this instructive pamphlet.

**Messrs. WM. BARTLEET AND SONS,** of Redditch, a firm established since 1750, are the manufacturers of all varieties of needles, and the house makes a specialty of surgical needles, safety pins, and exploring needles. The catalogue gives full particulars of many hundred varieties stocked, and any special sort of needle will be made to order. The surgical needles with spring eyes are now rapidly gaining in popularity owing to the ease with which they can be threaded. We have compared the prices of this firm with those charged by other houses and find that they are lower than the latter. Hospital authorities who require large supplies should write to Messrs. Bartleet for their special catalogue.

**Messrs. R. SUMNER AND CO., Ltd.,** Surgical Instrument Makers, of Liverpool, have won a high provincial reputation, and their specialities ought to be much more widely known in the metropolis than they are at

Michel's Suture Set.

present. The general practitioner will find their two-guinea aseptic minor operating case one of the finest in the market; with the additional instruments and the hypodermic outfit supplied to army medical officers, it is, at the

Sumner's Beaked Nail-nippers and Stitch-scissors.

price of three guineas, one of the best outfits that we are acquainted with. Full details are given in our advertisement columns. The firm lists special hollow back nail nippers, which ought to be provided in every operating room, specially curved improved tongue-forceps, binaural stethoscopes, throat forceps, and operating-room appliances. Among the last we may specially refer to the new aseptic dressing tables and the surgeon's muslinette coats and overalls, which are washable and can easily be rendered aseptic. This firm also supplies an improved Michel's Suturing Set—and we need not lay stress on the many advantages, so far as quick working is concerned, which the use of this method of suturing offers. The prices charged in the catalogue are very moderate and compare excellently with those asked by other firms. The instrument trays and tables listed by this firm are of a very solid yet light construction, and admirably adapted for hospital use. Authorities of cottage hospitals will find Messrs. Sumner's catalogue full of interesting details, and well worthy of study.

**Messrs. MAW, SON AND SONS,** 7-12 Aldersgate Street, E.C., draw special attention to their new instruments for use in gynæcological work, especially the forceps for clamping the vagina in a case of a total Wertheimer's

New Gynæcological Clamps.

operation. This firm lists a large number of special instruments, especially mouth and throat instruments, and stocks a variety of hospital fittings and furniture. Its catalogues will well repay attention, although the institutional worker will even do better by calling at the premises and inspecting the assortment of appliances, always on view in the show-rooms. Instrument racks and cabinets, theatre equipment, especially in operating-tables and accessories, electric apparatus, and furniture for special departments are made by the firm. The manufacture is in every way reliable, and the furniture keeps its appearance and its usefulness for, one is almost inclined to say, a lifetime. But it is essential that hospital authorities should realise that no appliance or apparatus can last for an indefinite time; all need renewal or renovation after a long working period. Those made by this firm, however, can be relied upon to last as long and to bear as hard usage as any manufactured in this country.

**Messrs. J. H. HAYWARD, Ltd.,** of Castle Gate, Nottingham, and 16 Silver Street, Wood Street, London, are surgical mechanicians whose Crepe Velpeau Stockings and Bandages have already been favourably noticed in these columns. One of the best modifications of elastic surgical stockings is listed by this firm; the Premier Adaptable Stocking is without rubber threads, porous and perfectly ventilated, and therefore much more economical in the long run than the ordinary surgical stockings supplied. It is specially suitable for warm climates.

**Messrs. E. LEITZ,** of Wetzlar, supply all varieties of optical instruments, microscopes, and accessories. The London branch of the firm is at Oxford Street, whence all information can be obtained. Leitz instruments have won a recognised reputation in the microscopical world, and the institution or practitioner that possesses a Leitz instrument can be assured that it or he possesses the best apparatus that can be bought. "The Microscope and Some Hints on How to Use it" is the title of an instructive little booklet which the firm supplies to clients; it gives full details regarding the construction and use of the now so indispensable adjunct to hospital and post-graduate research work.

The list supplied by the **HOLBORN SURGICAL INSTRUMENT COMPANY, Ltd.,** of 26 Thavies Inn, W.C., abound with novelties of which we can only notice a few. There is the apparatus for "606" as used at the Lock Hospital, and the modified "Holborn" all-glass syringes, which are particularly suitable for use in general practice. The most interesting announcement in the catalogue, however, is the series of pages dealing with apparatus for the Bier treatment. This firm lists all the suction cups, hot air chambers and tubes required for the hyperameic treatment, and the practitioner who wishes to try this method should communicate with the company and obtain quotations—which, we may add, are extremely reasonable. The firm has already a high reputation for the excellence of its appliances. The models of ward and hospital furniture listed are all interesting—some of them are strikingly ingenious patterns, which will appeal to those who have been used to the older and more clumsy models which are still to be found in many institutions.

A large number of interesting novelties are figured in the lists of **Messrs. MAYER AND MELTZER,** of 71 Great Portland Street, W. Among their new throat and nose instruments are various new forms of enucleators, tonsil compresses, plugging and tongue forceps. This firm also supplies the apparatus required for the operation of lymphangioplasty, for continuous saline infusions, for endoscopic examinations, and for various modified methods of spinal and general anæsthesia. The Roth Draeger mask which is now so universally used in Continental hospitals, and the Roth Draeger apparatus, which should be in every hospital, are supplied by this firm. The new modifications of old instruments, the many novelties for the use of the school doctor and the insurance examiner, together with the various modifications of institutional apparatus, are all fully described in the illustrated lists issued by the firm.

The Roth Draeger Inhaler.

**J. NESBIT-EVANS AND CO.,** of Birmingham, supply the "Nesbit-Evans Hospital Bedstead," which has been adopted as the ward bedstead by the Sheffield, Edinburgh, and Glasgow infirmaries. The new models of this bedstead show many improvements which will appeal to hospital workers: they are furnished with patent wheeling-out arrangement and with an inclining stand for altering the position of either end of the bedstead—a much-needed improvement which does away with the clumsy foot-blocks still so often seen in hospital wards. The strength and simplicity of the bedstead make it one of the best models for the institutional administrator to consider.

**Messrs. VINCENT WOOD,** of 4 Albion Place, Blackfriars, are the manufacturers of the well-known "Eureka" brand Crêpe Velpeau bandages, which are rubberless, porous, washable, and therefore sterilisable, and favourites wherever they have once been used. We have already drawn attention to the merits of this bandage, and those who have tried it will fully bear out our verdict that it is one of the best on the market. Messrs. Wood also make trusses, belts and hosiery, and cater specially for hospitals.

The neatly got-up catalogue of **Messrs. P. BEIERSDORF AND CO.,** of 28 Idol Lane, contains an amazing number of notes with regard to medicated and other plasters; adhesive plasters are a speciality of the firm which makes the well-known and deservedly popular Leukoplast. Leukoplast is one of the strongest and most reliable of adhesive plasters we are acquainted with; as a strapping it is superior to the home-made article, being much more cleanly and durable. Its use will be found to be an economy, since it does away with multitudinous bandages. We have ourselves used Leukoplast and have seen it in use in many English and foreign hospitals and have always found it extremely reliable owing to its strength, durability, cleanliness, ease of application, and utter freedom from irritating after-effects. The great point in its favour is the readiness with which it can be removed. Its non-irritability is another, for it enables the plaster to be applied to the most delicate skins (for instance, in strapping umbilical pads in babies, or in cases where irritation of any sort is particularly undesirable, as in strapping applied to paralytic patients). The adhesive power of these rubber plasters is immense; it must be tried to be believed. In cases of fracture of the femur, extension can be made by using Leukoplast, and indeed the manifold uses to which the plaster can be put need a special article for their enumeration. The firm also supplies porous plasters, tooth paste and medicated soaps.

Note.—Particulars of all the apparatus mentioned in these notes and of the various items referred to in our advertisement columns may be obtained on application to the firms mentioned.

## Figures and Tables

**Figure f1:**
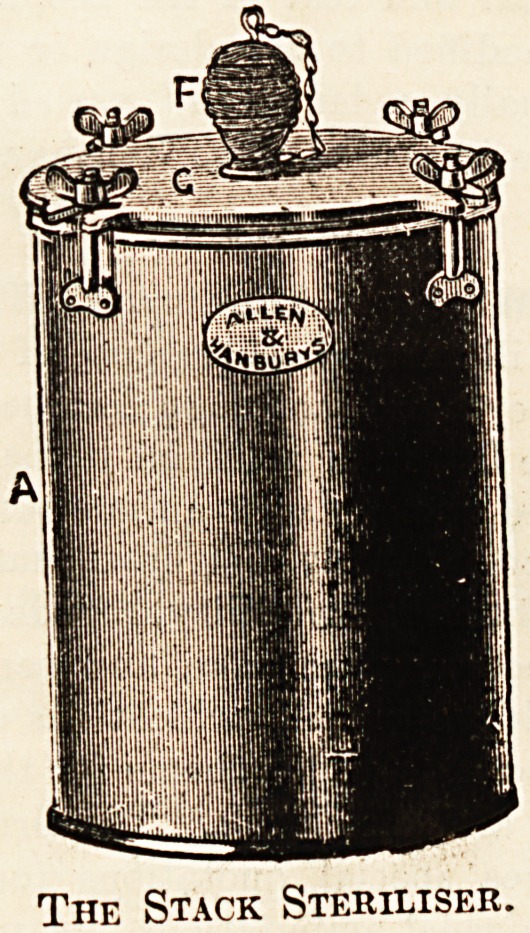


**Figure f2:**
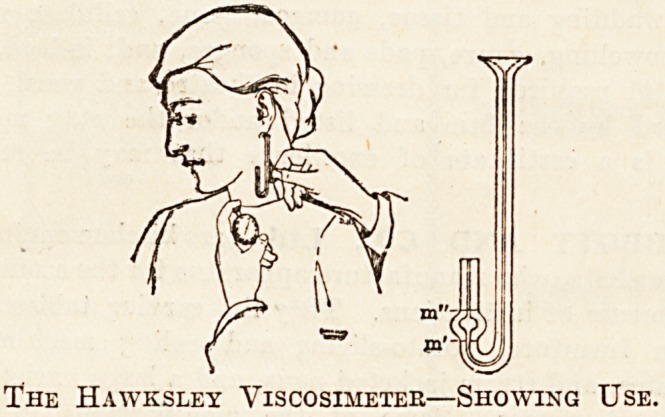


**Figure f3:**
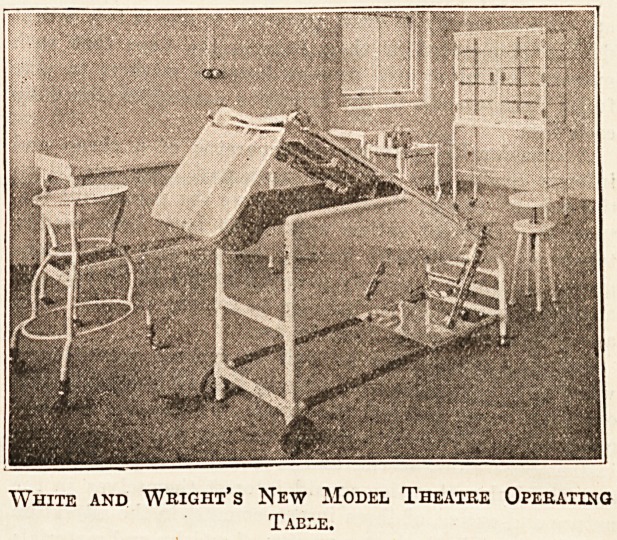


**Figure f4:**
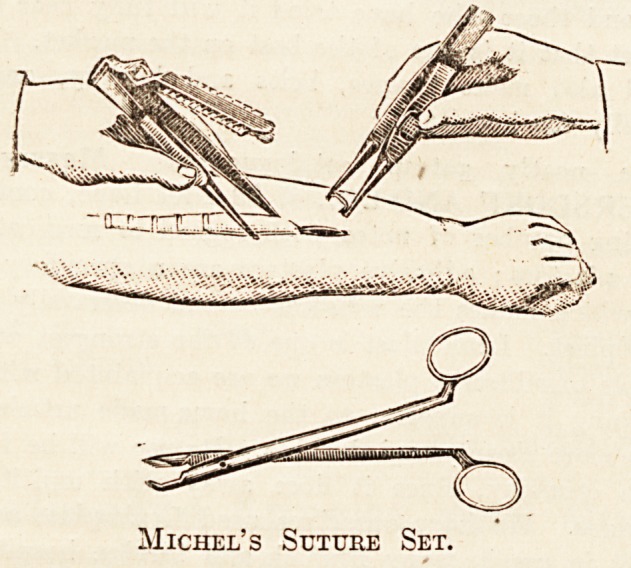


**Figure f5:**
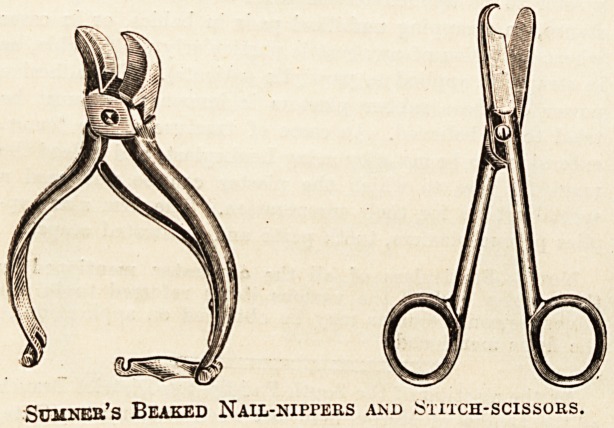


**Figure f6:**
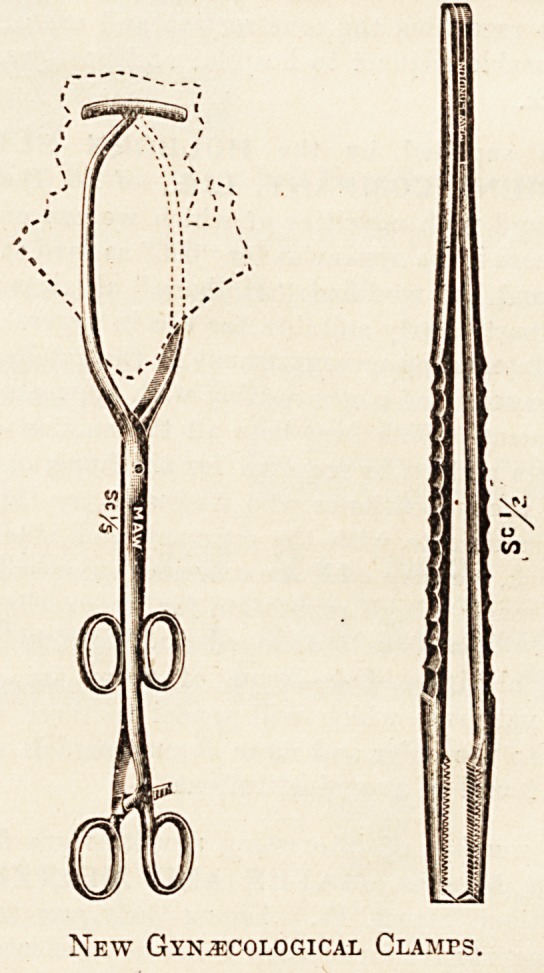


**Figure f7:**